# Neck Reflex Points: A New Clinical Test? Prevalence in Two Cohorts and Its Covariates

**DOI:** 10.3390/diagnostics14192185

**Published:** 2024-09-30

**Authors:** Stefan Weinschenk, Axel Gerhardt, Christine Wibmer, Thomas Strowitzki, Manuel Feisst

**Affiliations:** 1 Department of Obstetrics and Gynecology, Women’s Hospital, Heidelberg University, 69120 Heidelberg, Germany; 2 Department of Gynecological Endocrinology and Fertility Disorders, Women’s Hospital, Heidelberg University, 69120 Heidelberg, Germany; 3 The Heidelberg University Neural Therapy Education and Research Group, 69120 Heidelberg, Germany; 4 EbIM-Research Group, Unit for Complementary Medicine, Center for General Medicine and Evidence-Based Methods, Sigmund Freud University Vienna, Freudplatz 3, A-1020 Vienna, Austria; 5 Institute of Medical Biometry (IMBI), Heidelberg University, 69120 Heidelberg, Germany

**Keywords:** head and neck pain, muscular trigger points, neuro-reflectory changes, neural therapy, observational study

## Abstract

**Background**: Neck reflex points (NRPs) are 2 × 6 potentially tender areas of the neck, denominated NRP-C0 to NRP-C7. They are different from muscular trigger points and become tender in response to chronic trigeminal irritation. NRP examination has a high inter-rater reproducibility. We investigated the prevalence of NRPs in two populations to investigate their usefulness as a clinical test for trigeminal irritation. **Methods:** In total, 165 patients with chronic pain and 431 students were examined for NRP tenderness using a three-level pain scale: absent pain (PI = 0), mild tenderness (PI = 1), or marked tenderness (PI = 2). **Results:** In patients, we found more tender NRPs than in the student group (*p* < 0.001), and on the left side, more tender NRPs were found in NRP-C0–C4. Left and right NRPs appeared independently (kappa 0.1–0.4), except for NRP-C7 (kappa 0.55). Females had more tender NRPs (*p* < 0.001). Tenderness was independent of age, BMI, and pre-existing diseases. **Conclusions:** NRP tenderness occurs more frequently in patients than in students, independent from potential covariates. Our results, together with previous findings, support the use of NRP examination as a clinical test for chronic silent inflammation of the trigeminal region. These data provide a base for further studies investigating correlations of NRPs with clinical findings.

## 1. Introduction

**Figure 1:**
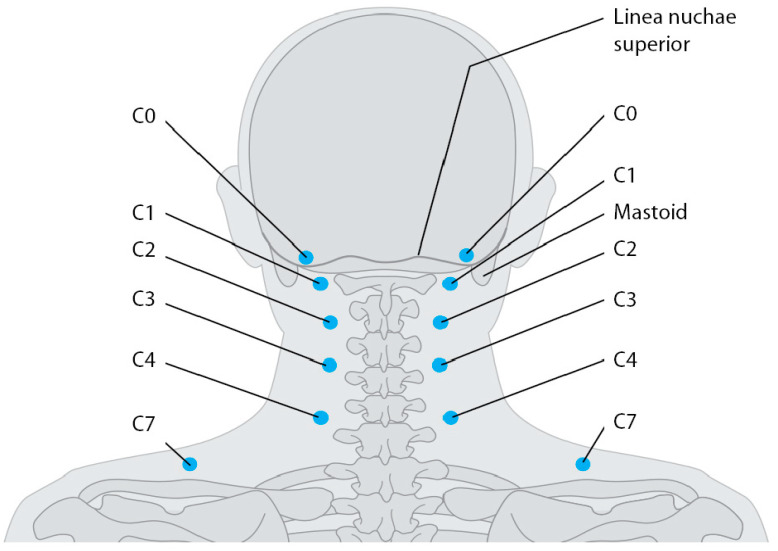
Neck reflex points NRP-C0–NRP-C7, according to [2,3].

Neck reflex points (NRPs) are twelve distinct tender areas located in the cervical neck and shoulder region, with six on the left and six on the right side [1], as illustrated in Figure 1. They were initially described and labeled as NRP-C0 through NRP-C7 by E. Adler [2], a Spanish dentist. In Central Europe, they were called “Adler–Langer–Punkte” [3]. To our knowledge, there are no clinical data on their relevance as signs and symptoms of remote disturbances such as silent inflammation, except in our own data [4].

In previous studies, we observed a high inter-rater reliability of NRP between two blinded examiners, with a kappa of κ = 0.80 for the left side and κ = 0.74 for the right [5]. The examiner’s experience level had no influence on detection rate and localization, suggesting that even for a beginner tender NRPs are easily examined, stable signs of remote disturbances [5]. The “subjective” rating of NRP tenderness by the examined has proven to be a valid measure. This is in contrast to investigations based solely on the palpation results of the examiner, such as higher stiffness and rigidity of the NRP, which did not reveal sufficient reproducibility [6].

The pathophysiological and neuroanatomic base of NRPs has not been clarified. NRPs are different from muscular trigger points (MTrPs), in that MTrPs are defined by characteristics such as referred pain, twitch phenomenon, and jump sign [7], none of which is found in NRPs.

Presumably, NRPs become symptomatic following a chronic irritation of the trigeminal nerve [1], e.g., in dental, sinusoidal, and pharyngeal chronic inflammation. In fact, tenderness of the NRP-C7 point is correlated with chronic pharyngitis [8]. We could demonstrate that the tenderness of this NRP (NRP-C7 in the trapezius region) was significantly reduced by injections of local anesthetics into this remote pharyngeal area [4]. These findings suggest a level-specific reactivity of NRP to chronic trigeminal irritation. Neuroanatomical and clinical data (e.g., in patients with headache) support the hypothesis of a trigeminal–cervical interaction [9,10,11,12,13]. Thus, the appearance of tender NRPs may be a sign of chronic silent irritation of the respective trigeminal area.

To clarify the possible significance of NRPs as a clinical test, it is necessary to know their prevalence. Do patients with chronic pain disease have more tender NRPs than average people do? Does the prevalence of NRP tenderness correlate with biometrical factors, such as gender, body weight, or age? Do different NRPs appear independently from each other?

We performed an observational study on two different Caucasian populations: chronically ill patients of an OB/GYN practice from an outpatient pain clinic in Germany, and a cross-sectional population of second-year medical students from Heidelberg University Medical School. Both were examined for the prevalence of tender NRPs.

We hypothesize that NRPs are found in patients more than in students. This may correlate with the acquaintance of diseases throughout a lifetime, especially functional disorders. Together with previous findings on reproducibility [5] and therapeutic response [4] to therapeutic injections with local anesthetics, these epidemiological data of NRP prevalence can contribute to a deeper insight into the role of NRPs as a possible diagnostic entity. Our aim is to enlarge the database and covariates of this promising new clinical mean.

## 2. Materials and Methods

### 2.1. Individuals Examined in the Study

#### 2.1.1. Students

**Figure 2:**
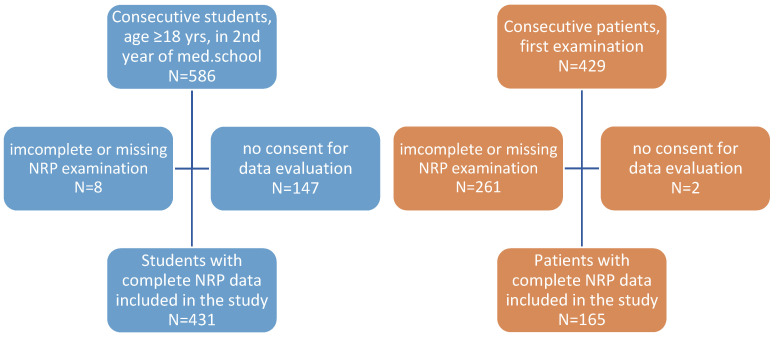
Selection of students (blue boxes) and patients (orange) according to the STARD criteria [14].

Students were routinely examined in a Pain Therapy course during their 2nd year of Medical School of Heidelberg University. They were recruited over the course of four consecutive semesters (summer 2006, winter 2007/08, summer 2008, and winter 2008/09), between July 2006 and February 2009, and served as an “average population” within our study. In total, 586 students were examined by the same lecturer demonstrating the NRP technique. In 8 students, the data sheet was incomplete (1.4%). Of the remaining 578 students, 431 (73.6%) gave their informed consent for anonymous data evaluation and could be included in the evaluation (Figure 2).

#### 2.1.2. Patients

A total of 429 patients with chronic pain visited the pain unit of an OB/GYN clinic in Karlsruhe, Germany, for the first time between January 2001 and December 2008. Patient examination usually includes an NRP examination. In all, 261 patients had an incomplete or missing documentation of all 12 NRPs and were excluded from the data collection (Figure 2). In 167 patients (39.2%), a complete NRP documentation was available. Amongst these, 165 individuals (98.5%) gave their informed consent for pseudonymized data evaluation and were included in the study.

### 2.2. Medical Disorders

Most patients complained of more than one chronic disorder. Reasons to seek medical advice are listed in Table 1. Patients complained of chronic pain—about half of them complained about pelvic pain, as well as other pain disorders, such as back pain (70%). The sum of the percentages exceeds 100% due to the multimorbidity of the patients. None of them suffered from widespread or generalized pain, or from fibromyalgia.

### 2.3. NRP Examination

The investigation of NRPs of the cervical neck was performed by an experienced physician (S.W.). Examination of NRPs followed a standardized procedure described in the neural therapy literature [1]. Patients were asked to report exactly on tenderness upon palpation at both sides and on every NRP level.

**Figure 3:**
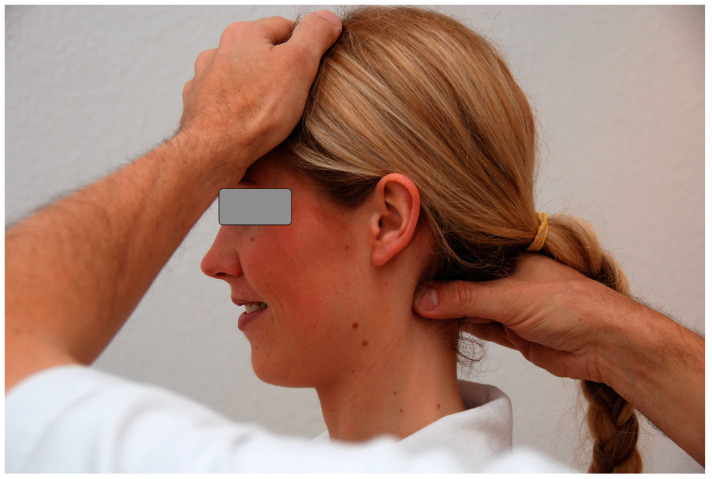
NRP examination, here: level NRP-C2 (© S. Weinschenk 2008). The examiner’s hand examines the neck soft tissue for tenderness at each level, from NRP-C0 to NRP-C7. The technique of the NRP examination is described in detail in [1]. Tenderness was described by the individual in three categories: absent (pain index, PI = 0), moderate (PI = 1), or strong (PI = 2).

An example of the examination procedure (level NRP-C2) is shown in Figure 3. NRP tenderness was qualified on a three-point scale according to Andersen and coworkers [15]. Absence of pain was defined as a pain index of PI = 0, slight tenderness was defined as PI = 1, and marked pain as PI = 2. The method is described in detail in [4,5]. It should be taken into consideration that the measure of interest in this study was *tenderness* as described by the patient, but not *rigidity* as felt by the examiner, which has been shown to have a low inter-rater reliability [6].

The age, gender, weight and height, and medical history of the patient group were obtained from the patient’s charts. The age and gender of the students were collected by the data sheets used for NRP documentation in the courses. The birthday of the patients was known exactly, whereas the age of the students was only given in year of birth; therefore, we input all students’ birthdays as taking place midyear.

### 2.4. Statistics

R version 4.2.0 was used for statistical analysis. Mean (SD) values were calculated for continuous outcomes, and absolute and relative frequencies were calculated for categorical variables. For summarized analyses (total pain and total pain l/r), the respective pain indices were summed up. Subgroup comparisons were performed using *t*-tests for continuous outcomes and chi-squared tests for categorical outcomes, respectively. To assess whether there is agreement between neighboring points, we calculated Cohen’s kappa coefficients and conducted McNemar’s tests. Correlations were assessed using Pearson’s correlation coefficient. In this exploratory study, statistical differences of *p* < 0.05 were considered to be significant in a descriptive sense.

## 3. Results

### 3.1. Characteristics of the Groups

In the student group, 61% of were female, and their average age was 22.5 years. In the patient group, 93% were female, the average age was 47 years, the BMI was 22, and the blood pressure was 127.4/82.1 mmHg on average (Table 2).

### 3.2. Average Number of Tender NRPs

The sum of tender NRPs in patients was 3.32 ± 2.29, in contrast to students, with 2.12 ± 2.07 (mild or marked tenderness, PI = 1 or 2), and 1.18 ± 1.28 versus 0.66 ± 1.07 with marked tenderness (PI = 2).

Counting the number of individuals with mild tenderness (PI = 1), marked tenderness (PI = 2), or both (PI = 1 or 2), we also found a significant difference between students and patients (*p* < 0.001); see Table 3.

### 3.3. NRP Tenderness Prevalence in Students and Patients

**Figure 4:**
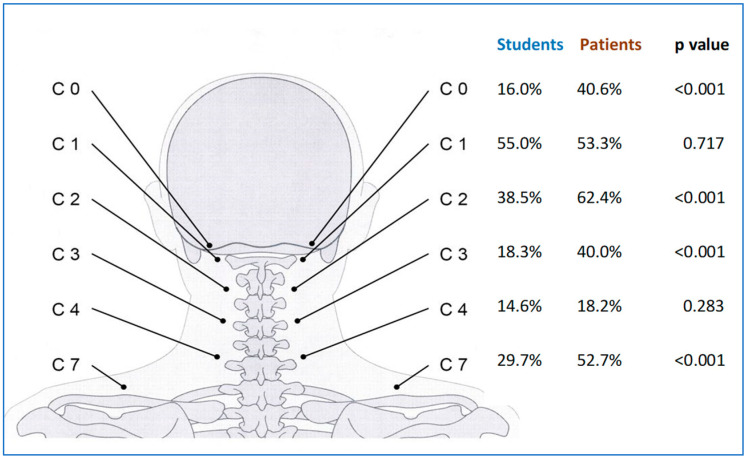
Percentage of mild and marked tenderness (PI = 1 or 2) in 165 patients and 431 students (sum of both sides and both tenderness levels). A significant difference between the two groups was found at the NRP levels C0, C2, C3, and C7.

We calculated the sum of the NRPs with marked or mild tenderness (PI = 1 or 2), as well as with marked tenderness (PI = 2) on every level. In four out of six NRP levels, the prevalence of NRPs was higher in patients than in the student group (Figure 4). The difference was significant in NRP levels NRP-C0, NRP-C2, NRP-C3, and NRP-C7 (*p* < 0.001).

The higher prevalence in patients was found for mild (PI = 1) and marked tenderness (PI = 2) as well: significant differences between students and patients on levels NRP-C0, NRP-C2, NRP-C3, and NRP-C7, but no difference on levels NRP-C1 and -C4. The results are displayed in Figure 4.

### 3.4. Side Prevalence of NRP Tenderness

We compared the prevalence of NRP tenderness of the left and right side in all individuals (see Table 4). We found significantly more tender NRPs on the levels NRP-C0, NRP-C1, and NRP-C2 on the left side (*p* < 0.001). In contrast, NRP-C7 tenderness was seen more frequently on the right side (*p* < 0.01).

Analyzing the side prevalence in both groups separately, we found significantly more tender NRPs in level C1 on the left side of the student group (46.7% vs. 25.8% for PI = 1 and 2, *p* < 0.001). In patients, there were more tender NRPs on the left side on level C1 (39.4% vs. 28.5%) and level C2 (43.7% vs. 33.3%), whereas on level C7, we detected more tender NRPs on the right side (46.1% vs. 39.4%), as well as for mild as for marked tenderness (PI = 1 or 2). The results are displayed in Table 5.

### 3.5. Left–Right Independence of NRP of the Respective Level

To analyze if there was an agreement between the left and right NRPs on the same level in students and patients, we calculated Cohen’s kappa coefficients (κ) and conducted McNemar’s tests for agreement for every level. According to Landis and Koch [16], no kappa agreement was denominated when κ was between 0 and 0.20, a fair agreement between 0.21 and 0.40, a moderate agreement between 0.41 and 0.60, and a substantial agreement between 0.61 and 0.80.

According to Landis and Koch [16], we found no agreement between the NRPs at the levels NRP-C0, NRP-C1, NRP-C2, and NRP-C3; a fair agreement at NRP-C4; and a moderate agreement at the level NRP-C7 (see Table 6). Following the scale proposed by Fleiss and coworkers [17,18], a fair-to-good agreement was found in NRP-C7 only. However, none of these agreements was significant.

The Cohen’s kappa coefficients (κ) and *p*-values according to the McNemar’s test for all NRP levels are listed in detail in Table 6: comparison of NRP tenderness between a single NRP and its respective counterpart on the other side with confidence intervals (in brackets). The kappa coefficient, κ, was calculated by the McNemar’s test and displayed with the respective *p*-values. Quantitative descriptions of correlations given in the last two columns follow the recommendation of [16,17,18].

### 3.6. Level Independence of NRP Tenderness

To evaluate a possible interdependence of neighboring NRPs, we calculated Cohen’s kappa for agreement of every NRP and its respective distal neighbors. We did not find an agreement in most of the comparisons between a tender NRP and its neighboring NRPs (Table 7). In only one comparison, C3 to C2, was there a significant agreement. It is called fair agreement according to the evaluation scale of Landis and Koch [16], or moderate according to the scale of Fleiss and coworkers [17,18]. For the other 29 of the total 30 comparisons of NRPs with their immediate or distant neighboring NRPs, we found no significant agreement. The kappa coefficients and *p*-values (McNemar’s test) for all NRP levels are displayed in Table 7.

### 3.7. Influence of Gender

**Figure 5:**
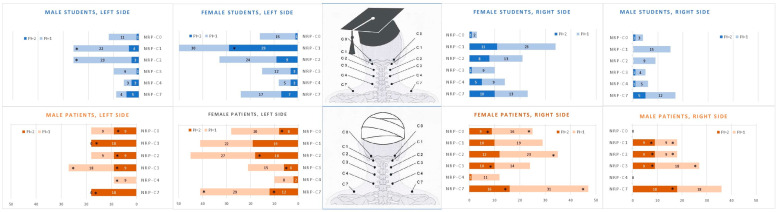
Prevalence of mild and marked NRP tenderness in both groups, N = 431 students and N = 165 patients (pain index, PI = 1, 2, or 1/2), at each NRP level of the left and right side, divided according to gender. * Difference is significant on a <0.05 level compared to the respective NRPs of the other group.

Gender may have a marked influence on the NRP prevalence. We calculated the distribution of tender NRPs separated to the gender. The results are displayed in Figure 5. In female students, we found more tender NRPs than in males in all levels but NRP-C2. Figure 5 shows the sum values of the left and right side of both genders in both groups in the respective level, NRP-C0 through NRP-C7.

We found a highly significant gender difference in patients between the group without tender NRPs (PI = 0) and the patient group with one or more tender NRPs: 79% of the patients with one or more tender NRPs (PI > 0) were female, whereas patients without NRPs were mostly males (38% females, *p* < 0.001).

### 3.8. Influence of Body Mass and Age

In students and patients, we obtained information on the age of the individuals, and in patients, we also obtained data on the body mass (BMI). Both characteristics may influence the prevalence of tender NRPs. Both variables showed no statistically significant difference between the PI = 0 and PI > 0 group (mean age, 30.5 vs. 32.2 (*p* = 0.182); mean BMI, 24.2 vs. 22.7 (*p* = 0.447).

### 3.9. Influence of Preexisting Diseases

**Figure 6:**
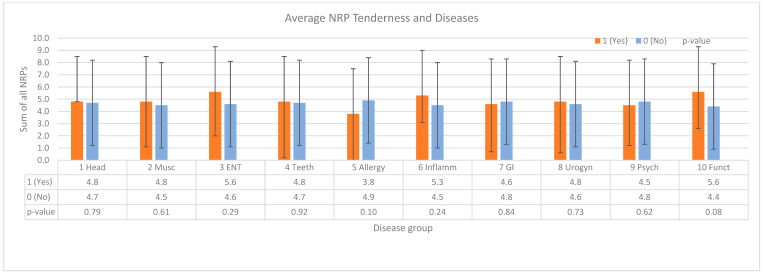
NRP tenderness with and without pre-existing diseases in N = 165 patients (pain index of PI = 1 or PI = 2). No significant difference in the average tenderness was found. Patient’s history of diseases was taken from the records of the patients. ENT, otorhinolaryngological disorders; Funct, functional disorders without morphological substrate; GI, gastrointestinal disorders; Inflamm, chronic inflammatory disease; Musc, musculoskeletal disorders; Psych, psychological disorders; Urogyn: urogynecological diseases.

NRP tenderness was claimed to be a sign of remote chronic disease of the trigeminal region, such as chronic irritation of the sinuses (NRP-C0 and NRP-C1), the teeth (NRP-C2 and NRP-C3), and the pharyngeal region (NRP-C4 and NRP-C7). We therefore investigated the correlation between NRP tenderness and pre-existing diseases taken from the patient’s history. Diseases were ordered in 10 groups; see Table 1 and Figure 6. There was no significant difference between individuals having the respective disease and not having it in and of the 10 groups, with *p*-values between 0.08 (group 10) and 0.92 (group 4); see Figure 6.

## 4. Discussion

### 4.1. Prevalence of NRP Tenderness

To our knowledge, this is the first report on the epidemiology of neck reflex points (NRPs). NRPs are six areas on each side of the cervical neck which may become tender, and which can be detected upon physical palpation [1].

NRP tenderness examination shows a high inter-rater reliability, independent from the examiner’s experience [5]. NRPs seem to be a clinical entity, different from muscular trigger points (MTrPs). They show none of the typical signs of MTrPs [7]. There is some empirical evidence that NRP tenderness can serve as an indicator for remote chronic disorders of the trigeminal region, i.e., in the visceral cranium [1]. They are to become tender as a reaction to a chronic irritation of the trigeminal area. NRP tenderness can be significantly reduced by therapeutic interventions: injections of local anesthetics to the pharyngeal region specifically reduce NRP tenderness [4]. This reduction may indicate a reduction in the chronic inflammation of the respective area in the visceral cranium.

Knowing a symptom’s prevalence is an important prerequisite to assessing the strength of a clinical test. We examined two independent populations for the prevalence of NRP tenderness: chronically ill patients, and students as a cross-sectional study group of young individuals.

**No influence of age**. Tender NRPs are thought to be signs of chronic inflammation in the trigeminal area, such as chronic sinusitis and pharyngitis. The incidence of chronic inflammations increases with age. We therefore postulated that the number of tender NRPs increases with age as well. We expected that patients “accumulate” tenderness of the NRPs during their lifetime. However, no age dependence of the NRPs was found, even when the data were adjusted to the factors “group” (patient vs. student) and “gender”. NRP tenderness seems to occur independently of the age of the individual. In contrast, a correlation was found in patients: they showed a significantly higher prevalence of tender NRPs than students (odds ratio, 3.4). We postulate that NRP examination discriminates against individuals with a low incidence of diseases from ill individuals seeking medical advice.

**No influence of body weight**. We expected a lower prevalence of tender NRPs in individuals with a higher body weight, assuming that body fat of the cervical neck may cover tenderness of NRPs. However, there was no correlation. We conclude that NRP testing can be applied independently of the BMI of the respective individual.

**Gender difference**. A higher prevalence of tender NRPs was found in females. The reasons are unclear. The difference may be based on differences of the female soft and connective tissue of their musculoskeletal system. It is also possible that the defined palpation pressure used in this survey, as well as in previous studies [19], is too low to examine males, possibly due to their higher muscular mass in the neck. But the gender difference may just as well reflect physiological gender differences in the NRP prevalence. Future studies investigating the clinical correlation of NRP tenderness will answer this question.

**Clinical correlation of NRP tenderness**. We do not know the clinical implications of tender NRPs. Except for the intervention study mentioned above [4], there are no data on NRP correlation with clinical findings of the visceral cranium. The Spanish dentist E. Adler, who first observed the NRP phenomenon [2], postulated a level-specific correlation of tender NRPs with diseases of the trigeminal forehead region. He ascribed NRP-C0–C1 to be correlated to chronic sinusitis, and NRP-C2–NRP-C3 to a dental chronic inflammation, such as an apical osteitis. Later, NRP-C4 and NRP-C7 were ascribed to chronic pharyngitis [1,3]. This assumption was supported by Uehleke et al. [8], who observed a high clinical correlation between pharyngitis and tenderness of NRP-C7. We did not find a correlation of NRP tenderness with pre-existing diseases. In fact, NRPs are intended to detect *inapparent* (otherwise unknown), pre-existing silent inflammations of the trigeminal area, but not *apparent* diseases. Therefore, this result of no correlation with apparent diseases is congruent with our hypothesis.

**Independence of contralateral NRPs**. Assuming the postulate of Adler that each NRP level indicates a specific and localized segmental disorder of the trigeminal region [2], we postulated that NRPs become tender independently from each other. We therefore examined the interdependence of NRPs with their contralateral counterparts. We did not find a correlation in NRP-C0 through NRP-C4 (kappa < 0.4). Only NRP-C7 showed a moderate correlation between left and right NRPs. NRP-C7 is claimed to be an area corresponding to the pharyngeal region. Chronic pharyngitis usually is a bilateral disease. This may reflect the agreement at the NRP-C7 level. In contrast, there was no left–right correlation between NRPs at all other levels, NRP-C0-C4. These NRPs are claimed to reflect chronic sinusitis and chronic dental osteitis. Both conditions may occur unilaterally, which corresponds with the findings of the independence of left and right NRPs.

**Independence of the neck levels**. We found no significant agreement between a certain NRP with its immediate, or with the next or other levels (kappa < 0.3). This is congruent with the clinical experience that local trigeminal irritations induced by a chronic inflammation, such as dental osteitis, occur independently from another inflammation site, e.g., frontal sinusitis. The findings of independence support the proposed specific relation of tender NRPs to a segment-specific disorder of the trigeminal region in the forehead.

### 4.2. Limitations and Strength

**Selection bias in the patient group**. Due to the location of the pain clinic within an OB/GYN unit, we examined mostly women in the patient group. In contrast, our student group was recruited from young individuals from the Medical School of Heidelberg University, with ~60% of the students being females. The average age and the gender distribution differed significantly between the two groups, meaning the two groups studied are not assimilable: we examined two different populations. As none of the students were patients in the OB/GYN pain clinic, there was no overlap between the groups, and all data could be calculated as independent samples. We found significant differences in the NRP prevalence between the patient and the student group. We conclude that patients are characterized by a higher prevalence of NRPs compared to controls, independent from age and gender.

**Limited information about the health status of the student group**. In the student group, no further factors (confounders) were evaluated; that is, we did not know anything about the true health status of the students. Based on the young age of the students, severe diseases may be less likely in this group, and, if few were present, their influence may have been diluted due to the magnitude of this cohort. In order to exactly describe the disease prevalence in a defined healthy population, cohorts with known health status should be evaluated in further studies. Nevertheless, the prevalence in a normal healthy population needs to be investigated in further studies. Based on the results of the current study, future case-number calculations can be performed.

**Reliability and objectivity of the NRP test**. The examinations were not double-checked by two independent examiners, so an examiner bias cannot be excluded. However, in a previous study, we could demonstrate a high inter-rater reliability even between an experienced and an inexperienced examiner [5]. We therefore assume that the individual examiner bias was low also in this investigation. It is noteworthy to mention that the base of the results is the pain expression by the patient only. Other investigators using the palpation findings of the examiner only did not find any reproducibility [6].

**Left-side predominance**. We found a higher prevalence of NRP tenderness on the left side in some of the NRP levels. The reasons for this are unclear. The difference may have been caused by the examination mode, in which the physician examines the individual with one hand standing laterally to the person. Other examination modes (standing in front of the patient) were described in the literature [1]. We decided to use this method because it was used in previous studies on NRPs [4,5]. Although examiners were trained to apply a defined pressure of 4 kp on each side, a palpation difference between the thumb and middle finger cannot be excluded. However, the predominance of the left-side tenderness, especially in female students, at the NRP-C2 level may be based on a currently unknown physiological correlation.

**Measurement scale**. The three-level scale (PI = 0, 1, 2) used in this survey may be discussed. Other scales such as the Nominal Analogue Scale (NAS) 0–10 may be used as well. The three-digit scale was introduced by Andersen and coworkers [15] for the examination of trigger points of the cervical neck. Here, as well as in previous NRP studies [4,5], this scale has revealed to be an easy-to perform, reliable, and easy-to-understand measure for both, patient, and examiner. From the patient’s point of view, it is much easier to answer than a 10-digit scale. From the physician’s view, a three-level distinction is completely sufficient when searching for a sign of chronic (silent) inflammation on a certain level. We therefore recommend maintaining this easy-to-use scale in further studies.

**Nomenclature**. The denomination of the NRP from “NRP-C0” to “NRP-C7” is historic and has been discussed in detail [5]. They are not necessarily correlated with the transverse processes of the respective cervical spine vertebrae. As in previous publications, we decided not to change these historic denominations, as this is of no relevance to the meaning of this clinical phenomenon. Apart from these nomenclature issues, the clinical relevance may be caused by a segment-based interrelation to the trigeminal region.

**Cutoff for positive findings**. Which result is clinically relevant for finding sites of chronic inflammation, moderate (PI = 1) or marked tenderness (PI = 2)? Using marked tenderness results in a lower number of positive results and allows for a better distinction between patients and students. As long as there are no correlation analyses with clinical findings, we suggest using the results of marked tenderness (PI = 2) as “positive” findings.

### 4.3. Is NRP Testing a Valid Clinical Test?

According to Guyatt [20], a good diagnostic test meets at least 5 of the 12 criteria listed below. NRP testing fulfils them in at least 5, and possibly up to 9, out of 12 categories, for instance, reproducibility [5], change in results following an intervention [4], and a moderate but not-too-low prevalence.

Applying the test criteria to NRP testing according to recent NRP publications and the present data of this study, we found that a minimum of 5, most probably of 9–12 criteria, were fulfilled (see Table 8).

## 5. Conclusions

**State of the Art**. The potential role of a single tender NRP as a sign of chronic illness in a specific region of the frontal head has been a matter of discussion for a long time [3,21]. Here, for the first time, we describe the prevalence of the NRPs in two different Caucasian populations, consisting of (a) patients with chronic pain and (b) young medical students. The epidemiological data on the tenderness distribution along the neck and the differences between patients and students, between males and females, and between left and right side of the cervical neck provide a base for the evidence-based use of NRP tenderness as a clinical test for silent inflammation.

**A potential clinical role of NRPs?** Calculating the specificity and sensitivity of NRP testing will be an important next step in evaluating the clinical relevance of tender NRPs. NRPs are assumed to be specific signs of chronic inflammation of the respective areas. In order to elucidate the clinical relevance, correlation studies combining clinical, laboratory, physical, and radiological findings with NRP examination results are to be performed. Our study creates a solid base for these investigations, including sensitivity, specificity, and likelihood ratios of this clinical test. Furthermore, our data can be used for case number calculations in future studies.

## Figures and Tables

**Table 1:** Complaints of N = 165 patients included in the study evaluation. Most patients suffered from several diseases (average, 4.3 entries per patient).

Complaints	Patients (N = 165)	Percent
Head and neck pain, including migraine	32	19.4%
Back pain and musculoskeletal disorders	113	68.4%
Otorhinolaryngological disorders (e.g., sinusitis)	25	15.2%
Dental disorders (e.g., dental pain)	53	32.1%
Allergy	20	12.1%
Chronic infection, chronic inflammation (e.g., chronic bronchitis)	40	24.2%
Gastrointestinal disorders (e.g., chronic gastritis)	53	32.1%
Uro-gynecological disorders (e.g., chronic pelvic pain)	80	48.4%
Psycho-social disorders (e.g., depression)	36	21.8%
Functional disorders (e.g., irritable bowel syndrome and fatigue)	45	27.3%

Note: The sum of the percentages exceeds 100% due to the multimorbidity of the patients.

**Table 2:** Summary of biometric characteristics of the patient and the student groups [1]. In 380 (88.2%) out of 431 students, complete gender and age information was available.

	Students	Patients	All Individuals
Number of individuals, N	431	165	586
Female (%)	231 (61%)	154 (93%)	385 (66%)
Age [years] mean ± SD	25 ± 3	47 ± 13	31 ± 13
BMI [kg/m^2^] mean ± SD	n.d.	22 ± 2.9	22 ± 2.9
Blood pressure [mmHg]	n.d.	127.4/82.1	127.4/82.1

BMI, body mass index; n.d., no data available.

**Table 3:** Percentage of individuals with tender NRPs (431 students and 165 patients).

	Students	Patients	*p*-Value
Number of individuals, N	431	165	
None (PI = 0)	98 (22.7%)	18 (10.9%)	
Mild (PI = 1)	168 (39.0%)	51 (30.9%)	
Marked (PI = 2)	165 (38.3%)	96 (58.2%)	<0.001
Mild or marked (PI = 1 or 2)	333 (77.3%)	147 (89.1%)	0.001

**Table 4:** Left–right prevalence of NRP tenderness in all individuals (N = 596). Differences between right and left NRP tenderness were significant on a level: * *p* < 0.05, ** *p* < 0.01, and *** *p* < 0.001.

NRP Tenderness (PI = 1 or 2)	Right Side	Left Side	*p*-Value
NRP-C0	52 (8.7%)	107 (18.0%)	<0.001 ***
NRP-C1	158 (26.6%)	266 (44.6%)	<0.001 ***
NRP-C2	121 (20.3%)	205 (34.4%)	<0.001 ***
NRP-C3	75 (12.6%)	95 (16.0%)	0.083
NRP-C4	69 (11.6%)	50 (8.4%)	0.028 *
NRP-C7	178 (29.9%)	150 (25.2%)	0.001 **

PI: pain index.

**Table 5:** Mild or marked NRP tenderness in N = 431 students and N = 165 patients (pain index, PI = 1, 2, or 1/2) at each NRP level of the left and right side. Asterisks denote that differences between students and patients were significant on a level: * *p* < 0.05, *** *p* < 0.001.

		Students	Patients	*p*-Value	Students	Patients	*p*-Value
NRP	PI	Left Side			Right Side		
NRP-C0	PI = 1	56 (13.0%)	120 (19.4%)	<0.001 ***	10 (2.3%)	24 (14.6%)	<0.001 ***
	PI = 2	6 (1.4%)	32 (7.9%)		4 (0.9%)	14 (8.5%)	
NRP-C1	PI = 1	117 (27.2%)	34 (20.6%)	0.204	84 (19.5%)	30 (18.2%)	0.242
	PI = 2	84 (19.5%)	31 (18.8%)		27 (6.3%)	17 (10.3%)	
NRP-C2	PI = 1	104 (24.1%)	43 (26.1%)	<0.001 ***	48 (11.2%)	36 (21.8%)	<0.001 ***
	PI = 2	29 (6.7%)	29 (17.6%)		18 (4.2%)	19 (11.5%)	
NRP-C3	PI = 1	48 (11.2%)	25 (15.2%)	0.019 *	30 (7.0%)	24 (14.6%)	<0.001 ***
	PI = 2	11 (2.6%)	11 (6.7%)		4 (0.9%)	17 (10.3%)	
NRP-C4	PI = 1	23 (5.4%)	13 (7.9%)	0.454	36 (8.4%)	17 (10.3%)	0.561
	PI = 2	11 (2.6%)	3 (1.8%)		13 (3.0%)	3 (1.8%)	
NRP-C7	PI = 1	59 (13.7%)	44 (26.7%)	<0.001 ***	65 (15.1%)	50 (30.3%)	<0.001 ***
	PI = 2	26 (6.1%)	21 (12.7%)		37 (8.6%)	26 (15.8%)	

**Table 6:** Left–right independence of NRPs in the student and patient group. The correlation is given as kappa values. The agreement is displayed according to the descriptions in the literature cited.

NRP Comp. with		Kappa κ [CI]	*p*-Value	Agreement Acc. to [16]
**Students**				
LiC0	ReC0	0.145 [0.026; 0.263]	0.017	None
LiC1	ReC1	0.195 [0.123; 0.267]	<0.001	None
LiC2	ReC2	0.164 [0.074; 0.253]	<0.001	None
LiC3	ReC3	0.188 [0.079; 0.296]	<0.001	None
LiC4	ReC4	0.402 [0.263; 0.540]	<0.001	Fair
LiC7	ReC7	0.532 [0.440; 0.625]	<0.001	Moderate
**Patients**				
LiC0	ReC0	0.173 [0.023; 0.322]	0.023	None
LiC1	ReC1	0.125 [−0.003; 0.254]	0.06	None
LiC2	ReC2	0.011 [−0.113; 0.135]	0.862	None
LiC3	ReC3	0.069 [−0.072; 0.210]	0.338	None
LiC4	ReC4	0.211 [0.024; 0.398]	0.027	None
LiC7	ReC7	0.572 [0.457; 0.687]	<0.001	Moderate

CI, 0.95 confidence interval; Comp., compared; acc, according.

**Table 7:** Comparison of NRP tenderness between the direct and further neighboring NRPs below (lines 1–5 for the neighboring NRPs and lines 6–15 for distant NRPs) in all individuals. κ (kappa) coefficient was calculated by McNemar’s test and given with the respective *p*-values.

			Right Side		Left Side	
	NRP	Comp. with	Kappa κ [CI]	*p*-Value	Kappa κ [CI]	*p*-Value
1	C0	C1	0.131 [0.060; 0.202]	<0.0001	0.151 [0.095; 0.208]	<0.001
6	C0	C2	0.179 [0.095; 0.263]	<0.0001	0.143 [0.071; 0.215]	<0.0001
10	C0	C3	0.222 [0.114; 0.329]	0.051	0.156 [0.071; 0.241]	0.130
13	C0	C4	0.134 [0.040; 0.228]	0.172	0.123 [0.036; 0.211]	<0.0001
15	C0	C7	0.178 [0.108; 0.248]	<0.0001	0.222 [0.141; 0.303]	0.0003
2	C1	C2	0.290 [0.212; 0.369]	0.0320	0.220 [0.155; 0.285]	<0.001
7	C1	C3	0.145 [0.069; 0.221]	<0.0001	0.072 [0.020; 0.123]	<0.0001
11	C1	C4	0.077 [0.011; 0.142]	<0.0001	0.056 [0.015; 0.097]	<0.0001
14	C1	C7	0.065 [−0.006; 0.137]	0.304	0.104 [0.043; 0.165]	<0.0001
3	C2	C3	0.285 [0.199; 0.370]	0.0003	0.286 [0.214; 0.358]	<0.001
8	C2	C4	0.122 [0.044; 0.200]	<0.0001	0.100 [0.045; 0.156]	<0.0001
12	C2	C7	0.062 [−0.003; 0.127]	0.093	0.127 [0.053; 0.201]	0.0002
4	C3	C4	0.268 [0.168; 0.367]	0.762	0.299 [0.197; 0.401]	<0.001
5	C3	C7	0.176 [0.106; 0.247]	<0.0001	0.162 [0.082; 0.243]	<0.001
9	C4	C7	0.190 [0.119; 0.260]	<0.0001	0.182 [0.107; 0.258]	0.012

CI, 0.95 confidence interval; Comp., compared.

**Table 8:** Application of test criteria described by [16,17,18] to NRP testing according to recent NRP publications and the present data of this study.

Test Criteria	Applied to NRP Test
Will the reproducibility of the test results and its interpretation be satisfactory in your clinical setting?	Yes [5]
Does the test yield the same result when applied to stable participants?	Yes [5]
Do different observers agree on the test results?	Yes [5]
Are the study results applicable to the patients in our practice?	Yes [4,5]
Does the test perform differently (different likelihood ratios) for different severities of disease?	Probably (these data)
Does the test perform differently for populations with different mixes of competing conditions?	Yes (these data)
Will the test results change your management strategy?	Yes
What are the test and treatment thresholds for the health condition to be detected?	Not known
Are the test likelihood ratios high or low enough to shift posttest probability across a test or treatment threshold?	Not known
Will patients be better off because of the test?	Not known
Will patient care differ for different test results	Probably
Will the anticipated changes in care do more good than harm?	Not known

## Data Availability

The data can be obtained from the corresponding author by reasonable request.
